# Advanced Fabrication Techniques of Microengineered Physiological Systems

**DOI:** 10.3390/mi11080730

**Published:** 2020-07-28

**Authors:** Joseph R. Puryear III, Jeong-Kee Yoon, YongTae Kim

**Affiliations:** 1 George W. Woodruff School of Mechanical Engineering, Georgia Institute of Technology, Atlanta, GA 30332, USA; jpuryear@gatech.edu (J.R.P.III); jyoon342@gatech.edu (J.-K.Y.); 2 Parker H. Petit Institute for Bioengineering and Bioscience, Georgia Institute of Technology, Atlanta, GA 30332, USA; 3 Wallace H. Coulter Department of Biomedical Engineering, Georgia Institute of Technology, Atlanta, GA 30332, USA; 4 Institute for Electronics and Nanotechnology, Georgia Institute of Technology, Atlanta, GA 30332, USA

**Keywords:** organs-on-chips (OOCs), microengineered physiological system (MPS), body-on-chips (BOCs), fabrication, microfluidic

## Abstract

The field of organs-on-chips (OOCs) has experienced tremendous growth over the last decade. However, the current main limiting factor for further growth lies in the fabrication techniques utilized to reproducibly create multiscale and multifunctional devices. Conventional methods of photolithography and etching remain less useful to complex geometric conditions with high precision needed to manufacture the devices, while laser-induced methods have become an alternative for higher precision engineering yet remain costly. Meanwhile, soft lithography has become the foundation upon which OOCs are fabricated and newer methods including 3D printing and injection molding show great promise to innovate the way OOCs are fabricated. This review is focused on the advantages and disadvantages associated with the commonly used fabrication techniques applied to these microengineered physiological systems (MPS) and the obstacles that remain in the way of further innovation in the field.

## 1. Introduction

The pharmaceutical industry has faced increased demand to generate human organ-mimicking platforms for rapid and efficient drug screening, as drug discovery and development are an extremely costly and time-consuming process. Based on recent data, the process takes anywhere from six to ten years and an estimated USD 161 million to USD 2.8 billion [1,2,3]. One of the primary contributing factors to the cost is the lack of experimental models to replace the expensive and timely process associated with animal studies [4]. Cultured cells have and are being served as one potential alternative to animal testing. They are widely used for drug discovery and development because they are easily available, easy to use, and have fairly low costs [5,6]. However, despite continual advancements made, approaches to culturing cells in vitro lack the ability to serve as a complete replacement of animal testing due to their inability to precisely mimic tissue-specific functions in living systems [4,7]. Existing model systems using conventional in vitro 2D cell culture configuration fail to reproduce the “active tissue-tissue interface” between the vascular endothelial cells and neighboring parenchymal tissues in which essential transport of fluids, nutrients, and immune cells are critical for the physiological function of living tissues and organs [4]. As a result, animal testing has been the primary method of choice for studying the potential effects drugs have on humans; however, animal testing is expensive, time-consuming, often controversial, and the results thus fail to properly model the effects on humans [8]. Approximately only 10%–15% of drugs entering clinical trials were approved for use by humans even following successful testing via animal trials [9,10,11,12].

**Figure 1:**
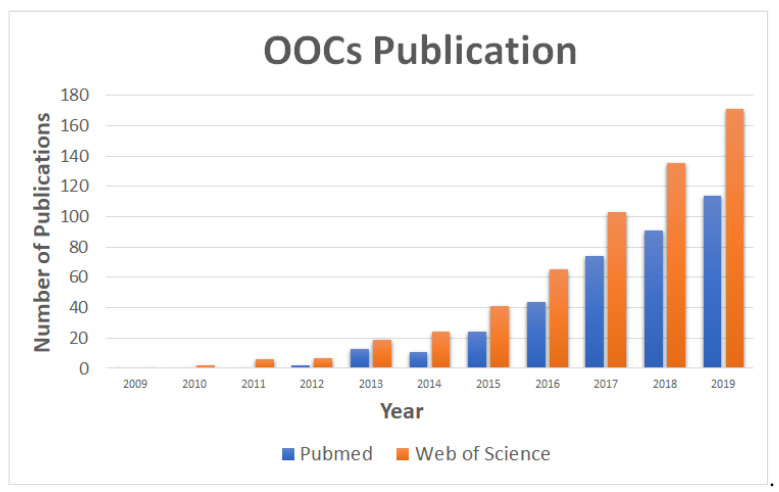
The publication history of organs-on-chips (OOCs) over the past decade according to Web of Science and Pubmed. “Organ-on-a-chip” and “Organs-on-Chips” were the keywords utilized.

With the creation of the first organ-on-a-chip (OOC) in 2010 by Huh et al., a new method was introduced which served as a potential replacement for drug discovery and development models and studies [4]. OOCs have been regarded as a device that incorporates multiple cells into engineered microfluidic channels or chambers to replicate critical aspects of the native tissue structure and function [6]. OOCs are a broad field that has garnered significant growth and attention over the past decade (Figure 1). The growth in OOCs has been exponential as new chips are developed and existing chips are innovated. One main advantage of using OOCs is the use of 3D features where cells grow in a condition more relevant to human physiology. The 3D aspects of OOCs provide the models with greater predictivity of gene and protein expressions, metabolic function, and physiological and functional readouts over other conventional two-dimensional (2D) models [13]. OOCs accomplish this by combining functional cells into 3D architectures, which can be performed by encapsulation in hydrogels or seeding onto scaffolds, to better simulate specific organ functions [13]. Since the first OOC was made up of immortalized cells, recent OOC devices have developed to incorporate primary cells and tissue biopsies, allowing for models to be created from the lung to the liver to the placenta [4,6,14,15]. OOCs have served as a promising complement to conventional models, allowing for more complex and dynamic interactions to fuel potential drug discovery and development [6]. As OOCs have made advancements, they can be subcategorized into three different groups: (1) modelling tissue barrier function within an organ (where lung-on-a-chip and blood-brain barrier-on-a-chip belong) [16], (2) modeling complex multifunctional functionality of a parenchymal tissue, and (3) modeling the systemic interactions between representative tissues and organs of the body (i.e., body-on-a-chip) [13]. Body-on-a-chip (BOC) are devices which allow multiple OOCs to be integrated together allowing for whole-body level responses [8,17,18]. The potential to combine numerous OOCs into BOCs could provide a human surrogate model for preclinical trials to further drug discovery and development. For example, a pumpless system that consists of 14 different chambers and 13 different organ/tissue mimics was developed [10]. Organs-on-chips (OOCs) studies over the past decade have been shown to have numerous advantages over conventional models and continue to serve as promising models which could eventually fully replace animal testing. They are used in a wide range of applications to explore and recreate the cellular behaviors that occur in organs and tissues in vivo. The technical knowledge required to design and fabricate OOCs and BOCs remains a primary limitation in the field [6]. This includes the fabrication process which is broken up into four components: (1) “a microfluidic chip”, (2) “2D/3D microtissues that are cultured in the chip”, (3) “components of stimulus loading to mature the microtissues” (e.g., electrical, mechanical, biophysical, growth factor, etc.), and (4) “sensors for monitoring the physiological behavior of microtissues and for results readout” [19].

A brief introduction to the various fabrication methods utilized to create OOCs can be found in Table 1. These methods will be elaborated upon later in this article to discuss the basic functions, advantages, and disadvantages of each. The fabrication of OOCs with high reproducibility for manufacturing remains one of the most critical steps to properly mimic the physiology of the target organ which can provide some specific environmental behaviors. During OOC development, it remains crucial to choose the fabrication technique best suited for the organ and experiment at hand. Key attributes to consider when choosing a method are: (1) considering the cells/tissues used which factors into the physical limitations and materials available to use (e.g., 3D printing strains the cells by inducing unnecessary shear stress, poly(dimethylsiloxane) (PDMS) has low bio-resistance due to the adsorption of organic solvents into the walls); (2) the technical knowledge required to use a method; (3) the cost and resources required; (4) time constraints and throughput requirements; and (5) the minimum feature size. With so many factors to consider when designing an OOC, choosing the proper fabrication technique is crucial and specific to each application. In this Review, we will discuss the existing fabrication techniques utilized to create OOCs and the advancements being made in each of these techniques.

## 2. Conventional Fabrication Techniques

Historically, the most conventional microfabrication technique utilized is the combination of photolithography and etching. While it is capable of producing numerous complex devices, it is highly limited by the materials available to use, with silicon or silicon-based glasses being the primary choice [20]. Silicon is a great choice for micro-electromechanical systems (MEMS); however, it lacks key attributes to be equally successful with OOCs [20]. Silicon and glass are chosen for MEMS devices because of their inertness, high strength, and thermal conductivity; however, their inability to allow gas to permeate makes them a poor choice for cell culturing [21]. With that in mind, the material should be limited solely by its own properties and not by the limitations of the desired fabrication process [20,22]. In addition to the limited material selection, photolithography has high cost associated with it, especially with sub-micrometer feature sizes [20,23]. One method of overcoming traditional photolithography limitations is using extreme ultraviolet lithography (EUV) which utilizes 13 nm light created from using a series of mirrors to focus EUV light emitted from an illuminated plasma [24,25]. The high cost of using EUV has limited it to rare applications (e.g., creating nanopillars) [26]; however, as smaller feature sizes are required to perform nanofluidics, it could be further developed for wider applications. Another popular variation on photolithography is the use of electron beam lithography (EBL). EBL utilizes a focused electron beam to scan over a mask-less surface. EBL eliminates the complications of utilizing a mask and allows for small feature sizes (e.g., less than 10 nm) [27,28]; however, EBL has an extremely low throughput due to the serial process [28]. EBL is being developed further to allow wider applications, but further limitations beyond the low throughput like high expenses continue to be a challenge [29]. Meanwhile, wet etching places limitations on potential materials because acids/bases can damage polymers [30,31]. However, track etching has proven quite useful in creating porous membranes made of poly(carbonate) (PC) and poly(ethylene terephthalate) (PET) for OOCs [30,31]. Wet etching consists of submerging the device into a chemical etchant (e.g., KOH) to remove material. Track etching uses heavy ions to target specific spots on a polymer [31]. This causes the polymer to degrade in these locations allowing for a shortened and more controlled wet etchant to complete the process [31]. Despite these negative attributes, these conventional methods have been applied to various OOCs (e.g., lung-on-a-chip, liver-on-a-chip, etc.) requiring multi-step processes and masks [19]. Inherently, the fabrication costs and time associated with developing the devices increase despite their limitation of only being able to fabricate the microfluidic chip [19]. This means that additional processes are still required to implement the other three stages described earlier to fully develop an OOC [19].

## 3. Advancements on Organ-On-A-Chip Fabrication Techniques

### 3.1. Laser-Induced Methods

Laser-induced methods refer to fabrication techniques that predominantly rely on the application of a laser. Various methods have been used for fabrication with one of the most popular being laser machining. For example, an eye-on-a-chip was created to study the formation of silicone oil droplets in the eye by laser engraving poly(methylmethacrylate) (PMMA) sheets [32]. Laser machining was also used to create a liver-on-a-chip where laser cutting and computer numerical control (CNC) was combined to fabricate the device to track the dynamics of mitochondrial dysfunction [14]. While laser machining itself is not widely applied to OOCs, other laser-induced methods have been used as well. In addition, an ultraviolet laser was used to perform photopatterning on gelatin hydrogels [53]. This method consisted of utilizing a UVA-light activated photosensitizer and a UVA laser engraver to perform ablation on the hydrogels, allowing for desirable patterns to be created for OOC applications. Traditional methods require mechanical molding of the hydrogel; while this laser-induced method allows for a mask-less high throughput patterning which shortens the typical fabrication process by 60% and allows for parallelization [53]. Meanwhile, a process was created that combines “ultrashort pulse laser-assisted chemical etching of glass, 3D laser subtractive glass printing, and carbon oxide laser-induced glass melting” to fabricate “freeform 3D microfluidic networks encapsulated in 3D printed glass macroscale objects” [54]. This method allows for a monolithic approach for fabricating 3D freeform encapsulated microchannels with 3D printed glass structures [54]. The hybrid process provides high precision at both the micro (tens of micrometers) and macro (several centimeters) scale in a simple and flexible monolithic process [54]. It also allows for controllable sealing of ports and chemical inertness compared to other fabrication methods which often rely upon poly(dimethylsiloxane) (PDMS) [54]. Laser machining has also been used to create patterned adhesive film-based microfluidic devices [55,56,57]. Multiple tapes and plastics (e.g., PMMA) are individually cut using laser machining and, subsequently, layered together onto a glass slide allowing for a rapid, low-cost fabrication [55,56,57]. Ultimately, the largest obstacle to overcome when attempting to use laser-induced methods is that they all require high familiarity with the fabrication process [58]. They remain complicated processes that are not easily utilized by beginners and often require expensive machinery to perform.

### 3.2. Soft Lithography

**Figure 2:**
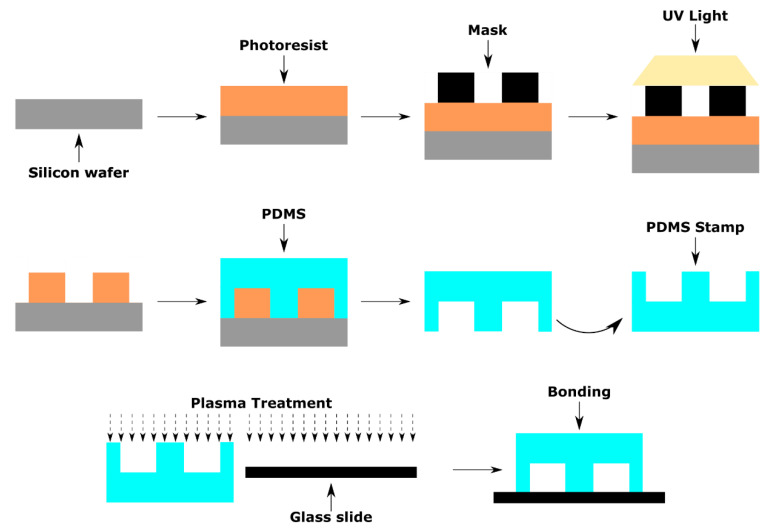
An example of replica molding using poly(dimethylsiloxane) (PDMS). The typical photolithography steps are taken utilizing photoresist, a mask, and UV exposure to create the master. The master mold is filled with PDMS and cured. Once cured, the PDMS is removed from the master and bonded to a glass slide for plasma oxidation.

The most common fabrication technique for OOCs is soft lithography [31]. Soft lithography molds soft elastomers instead of relying upon etching or deposition to create 3D structures. Soft lithography has numerous advantages including low cost, easy to use, and high compatibility with various materials [59]. The most widely used material when performing soft lithography is PDMS because it is transparent, biocompatible, gas permeable, easy to handle, and cost effective [60,61,62,63]. All of which are highly desirable traits of OOCs. As previously mentioned, other materials can be used in lieu of PDMS including polyurethane (PU) and polyimide [64]. One of the primary limitations of performing soft lithography is the low bio-resistance associated with materials like PDMS. The absorption of proteins, drugs, as well as in situ cell derivatives onto PDMS walls interferes with accurate analysis. In order to overcome this, new classes of elastomers have been developed called high fluorinated elastomers, which have better bio resistance and inertness than PDMS [65,66]. Another method of overcoming material property constraint is by mixing a polymer like PDMS with a curing agent at desirable ratios and curing them to adjust the mechanical properties. This method has proven extremely useful in adjusting the mechanical properties of PDMS to be used in numerous OOCs including heart-on-a-chip, liver-on-a-chip, lung-on-a-chip, and vascular-networks-on-a-chip [4,14,31,34,35]. The most common application of soft lithography is performed through replica molding [31]. Replica molding generally follows the following steps: (1) Using computer aided design (CAD) to create a pattern, (2) using photolithography techniques to develop a master, (3) filling the master mold with PDMS and curing it, (4) removing the PDMS from the master, and (5) bonding the PDMS to a glass slide and performing plasma oxidation (Figure 2) [59]. Plasma oxidation is essential to the process because it assists in the bonding process and converts the PDMS surface from hydrophobic to hydrophilic allowing the device to provide a better biocompatible environment for the cells to attach on, or mimic in vivo fluid interactions. This method was first utilized in OOCs with the development of the first lung-on-a-chip in 2010 to produce hollow microchannels [4]. Since then soft lithography has been widely applied to OOCs (e.g., lung-on-a-chip, liver-on-a-chip, tumor-on-a-chip, gut-on-a-chip, retina-on-a-chip, placenta-on-a-chip, brain-on-a-chip) [4,14,19,31,34,35,36,37,38,67,68,69]. However, soft lithography remains a multi-step process requiring masks, dedicated equipment, and familiarity with the technique [58]. In addition to these, soft lithography requires a lot of manual operations and can only be applied to one side of the device while the other side remains flat and smooth [31]. Inherently, these place constraints on the time, expense, and feature size [19]. Soft lithography remains most suitable for performing large scale production of the microfluidic devices and creating porous membranes for OOCs [31]. Additional components of OOCs such as microtissues, stimulus loading components, and sensors still require additional processes to complete the fabrication of OOCs [19]. As a result, soft lithography techniques have been further developed by exploring new polymers and combining soft lithography with other techniques to improve the fabrication process.

In order to overcome many of the PDMS material property limitations, thermoset alternatives have been used which are capable of generating more complex and in vivo-like structures [70]. One method of combining fabrication techniques was the combination of replica molding with “a novel method of structuring cell-laden hydrogels within microfluidic channels” [71]. This novel method used two PDMS molds and hydrogel injection to place two different hydrogels side by side within a microfluidic channel [71]. By combining these two, they were able to simultaneously provide fluid flow and control the cell structures which could not be accomplished with soft lithography alone [71]. The ability to generate multiple aspects of OOCs simultaneously is crucial for simplifying fabrication processes [29]. Another innovation was accomplished when developing a lung-on-a-chip which consisted of a poly(latic-co-glycolic acid) nanofiber membrane to perform anti-cancer drug screening [33]. The device was created by utilizing soft lithography for the top and performing electrospinning for the nanofiber membrane [33]. Perhaps the greatest development in soft lithography has occurred in a subfield known as nanoimprint lithography. This process consists of stamping a master mold into a material and hardening the material using some type of thermal, chemical, or optical curing process [72]. Nanoimprint lithography has great potential because it is a high throughput process with relatively low cost and can achieve sub-micrometer feature sizes [29]. As a result, large companies such as Canon have recognized its advantage over other fabrication techniques like EBL and have dedicated investments towards further advancing nanoimprint lithography [73]. New techniques in nanoimprint lithography such as roll-to-roll (R2R) nanoimprint lithography continue to show promise in increasing the throughput of the process [74]. The R2R method is dependent upon a flexible mold shaped into a roller that continuously rolls over a substrate resulting in a significant change in throughput [74]. A basic example of how this method works is a rolling stamper which consists of placing the stamp upon the material and rolling it 360° in order to complete the graphic. However, the improvement of the throughput comes at a cost of the minimum feature size attainable [74]. Nonetheless, soft lithography remains the foundation of fabricating OOCs.

### 3.3. 3D Printing

3D printing consists of layer-by-layer fabrication capable of producing various complex 3D structures allowing for rapid prototyping [19]. As a result, 3D printing has become one of the most promising fabrication techniques to produce OOCs [19]. The advancements in the resolution and speed of 3D printing technology over the past decade have made fabrication of microfluidic devices simpler [58]. Unlike the previously mentioned fabrication techniques, 3D printing also has the potential to print all the necessary components of an OOC not just the microfluidic device. 3D printers can embed tissue scaffolds into the microfluidic device because it can use a wide range of different materials including biomaterials (e.g., living cells) [58]. In theory, the use of a 3D printer for the fabrication process consolidates it all onto one fully automated machine, reducing the fabrication time and easing the replication process [21]. The simplified replication process is a result of 3D printing relying highly upon standardized CAD programs for the design process allowing for easier sharing/transferring across lab groups [58]. The potential of 3D printing OOCs is best described as a “’fail fast and often’ strategy in which early and rapid empirical feedback is used to guide and accelerate device development” [58]. However, the application of a 3D printer to simplify the process requires significant optimization as constraints such as the heat and pressure to print both polymers and bioinks/cells are contradictory, as the requirements necessary to print polymers would cause damage to the bioinks/cells. As a result, 3D printing hardware must be developed further in order to overcome these constraints. A brief overview of the various 3D printing techniques and their applications can be found in Table 2. In this review, bioprinting is considered separately from stereolithography, extrusion-based, and inkjet printing methods in this paper. While bioprinting is an extension of these methods, it is considered independently as the discovery of bioprinting spurred further innovations in the fields of 3D printing and microengineered physiological systems (MPS) due to its distinction of incorporating live cells in the ink.

#### 3.3.1. Stereolithography

**Figure 3:**
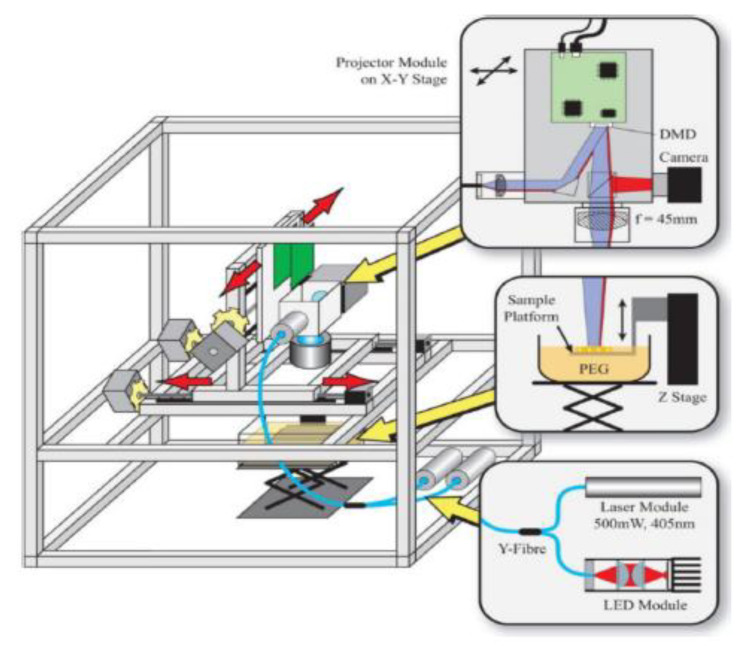
A stereolithography 3D printer (reprinted with permission from Lee et al.; copyright (2015) [81]). The translational stages and projector system are supported by an aluminum frame [81]. A Y-fibre is used to combine the light from a UV laser and a red LED [81]. This results in illuminating a Digital Micromirror Device (DMD) projector [81]. A lens projects the image of the DMD onto a sample platform immersed in the photopolymer (e.g., PEG) [81].

A typical 3D stereolithography printing process consists of using an ultraviolet laser to cure fluid resin layer-by-layer as a sweeping blade places a new layer of uncured fluid resin onto the cross section; this process is repeated until the desirable structure is completed (Figure 3) [58]. This technique was initially introduced in 1986 by Charles W. Hull who described it as an additive manufacturing process dependent upon the use of a printhead with a reservoir using bioink [75]. Since its inception, stereolithography has become the most widely used 3D printing technique and highly commercialized [58]. The main advantage of Stereolithography is the high resolution and ability to produce a “microscale fluidic” chip, the first element of OOCs described earlier [19,58].

Furthermore, stereolithography has a low cost and continual innovations have resulted in the printers being more affordable, faster, and smaller than ever before [58]. The primary reason stereolithography is faster is because the system only moves in the z-direction compared to other nozzle-based printers which utilized movement in the x-, y-, and z-direction [76]. This method also avoids any potentially harmful shear stress on the cells and allows for a wide range of biomaterials to be used since it does not require highly viscous fluids like other nozzle-based printers [76,77]. However, stereolithography struggles to print microtissues as well as other 3D printing techniques and has a lower resolution (e.g., 100 μm) compared to other techniques like soft lithography [19,58].

Stereolithography is constantly being innovated for further use in microfluidics and more specifically in OOCs. The stereolithography technique was sped up even further by creating a layer-less process using an oxygen-permeable surface which prevents the material from curing at the surface, eliminating any pull-off steps required at the end [78]. Further research has also been explored in custom bio-resins for use in stereolithography to enhance the process [29]. For example, a perfusion network was created that encapsulates a print and is permeable via a high weight poly(ethylene glycol) diacrylate (PEGDA) resin [41]. Stereolithography has also been combined with other fabrication methods to be more effective. A multicellular spheroid culture device was created using stereolithography and Polyjet commercial printers [42]. Meanwhile, double digit micrometer features have been achieved using a custom stereolithography printer in conjunction with a custom resin resulting in ~20 μm features [79]. This method was also capable of producing dynamic systems like pumps and valves [79]. Implanted porous membranes have also been shown to be created using a sequential stereolithography printer by switching between two resins [39]. A vascular network was also fabricated using carbohydrate glass via a stereolithography printer [80]. Stereolithography has also been combined with soft lithography to fabricate a lung-alveolus-on-a-chip to study inflammation induced thrombosis [40].

#### 3.3.2. Extrusion-Based

**Figure 4:**
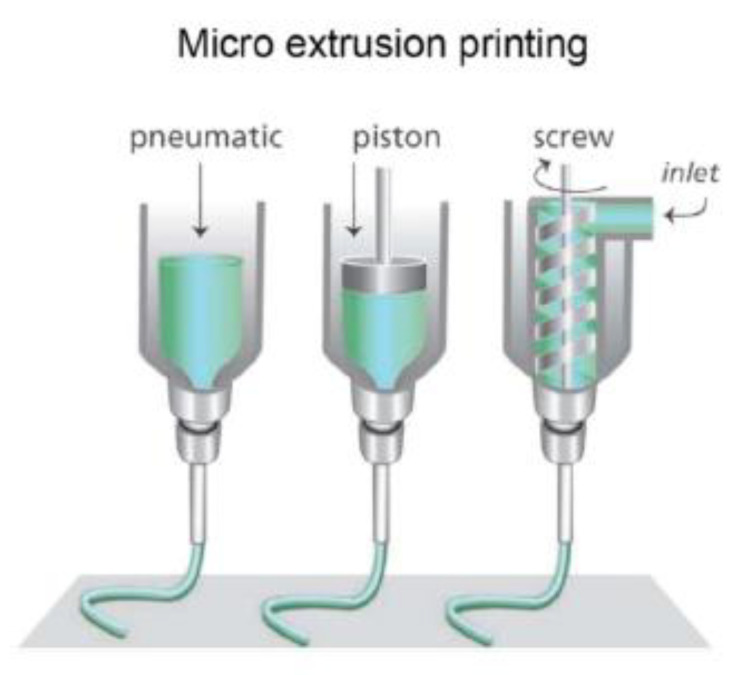
Three of the most common extrusion-based nozzles used for 3D printing (reprinted with permission from Malda et al.; copyright (2013) [86]). Hydrogels with suspended cells are inserted into the syringes and extruded out using either pneumatic, piston, or screw driven methods [86].

Extrusion-based 3D printing was originally developed in 2002 and typically consists of a heating element to melt the material and allow for it to be extruded out via a pneumatic or mechanical driven device (Figure 4) [82]. Extrusion-based printing, like stereolithography, is predominantly used for fabricating the first element of OOCs (e.g., the microfluidic device) [19]. However, innovations in micro-extrusion with bioprinting has made it possible to also print the second element of OOCs (e.g., microscale tissues) as described earlier [19]. In order to overcome the limitations associated with printing microtissues, it the use of both stereolithography and extrusion-based techniques has been suggested to produce the microfluidic chip and microtissues, respectively [19]. However, even since this initial suggestion in 2017, to the best of our knowledge, no such combined technique has been accomplished [19]. Extrusion-based printing’s primary advantages lie in its ability to print highly viscous bioinks and to continuously print. As a result of these advantages, extrusion-based printing can use a wide variety of thermoplastics (e.g., acrylonitrile butadiene styrene (ABS), polylactic acid (PLA), polyamide, etc.) [83]. In addition to this, extrusion-based printing has low costs, is easy to fabricate with, and is faster than conventional methods [29]. In 2017, an OOC with the ability to measure the contractile stress of cardiac microtissues was fabricated [84]. This was accomplished by creating six custom inks capable of integrating soft strain gauge sensors into the cardiac microtissues [84]. It has also been used in conjunction with replica molding to fabricate a bone-on-a-chip to study breast cancer cells [47].

#### 3.3.3. Inkjet

**Figure 5:**
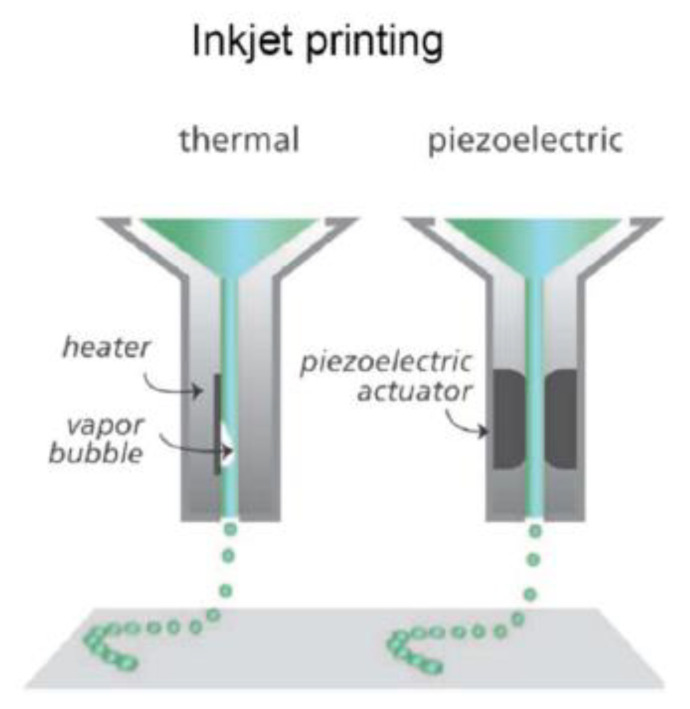
Two of the most common inkjet nozzles used for 3D printing (reprinted with permission from Malda et al.; copyright (2013) [86]). Thermal printing vaporizes small volumes of the ink to generate the necessary pulse to expel the droplets [86]. The piezoelectric method uses a direct mechanical pulse to generate a shockwave to expel the droplets [86].

Inkjet printing is based on a layer-by-layer contactless procedure of placing picolitre droplets which are cured by an ultraviolet light (Figure 5) [58]. Inkjet printing for microfluidics was created in 2003 and patented in 2006 [85]. Its ability to fabricate the first two elements of an OOC (e.g., the microfluidic chip and microtissues) makes it a versatile tool for fabricating OOCs [19]. Inkjet printing has many advantages including low cost, high quality, high accuracy, a fast build time, and the ability to function with numerous materials [58]. The advantages of inkjet printing can be furthered thanks to its simplicity which allows multiple printheads to work together to fabricate a device [75,77,85]. This method of 3D printing has not been as widely explored and applied as stereolithography and

Extrusion-based printing. Nevertheless, its technique has been applied to fabricate a chip to study cancer cells in a co-cultured microfluidic environment [50]. Inkjet printing has also been applied to a liver-on-a-chip to study drug metabolism and diffusion while showing the ability to utilize multiple cell patterns on a single chip [49].

#### 3.3.4. Bioprinting

**Figure 6:**
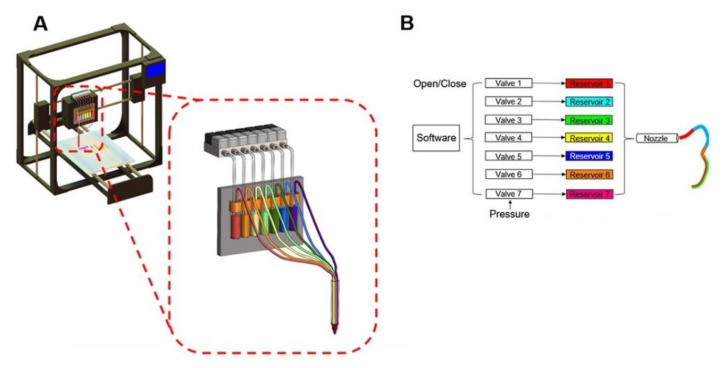
An example of a bioprinting setup (reprinted with permission from Liu et al.; copyright (2016) [99]). The design of a “digitally tunable continuous multi-material extrusion bioprinter” consisting of a “seven-channel printhead connected to reservoirs that are individually actuated by programmable pneumatic valves” [99].

The newest innovation in the field of 3D printing for OOCs is bioprinting. Bioprinting is an extension of the previously mentioned 3D methods. Due to its versatility and assembly-free process, bioprinting has been applied to various existing fabrication processes of OOCs [19]. Bioprinting consists of printing cells and biomaterials into scaffolds and structures using existing 3D printing methods (Figure 6) [58]. Various printer heads can be used in this process for printing different materials, allowing for complex multi-cellular structures to be formed [58]. While other processes are limited to producing only the first element of OOCs (e.g., the microfluidic device), bioprinting is focused on the second element (e.g., the microtissues). As a result, it has been widely applied to the field of OOCs. Bioprinting has been used to create hydrogel microchannels to serve as a vascular network [51,58]. One of the biggest challenges of OOCs is fabricating cardiac devices due to the complexity surrounding the myocardium; as a result, most fabrication techniques are incapable of creating it [87]. However, 3D bioprinting with the use of custom bioink has made it simpler and more feasible [87]. It has advanced the heart-on-a-chip by showing increased potential in accurately generating the mechanical properties of the heart [42,49]. This enhanced ability to reproduce more accurate and in vivo-like heart-on-a-chip devices was displayed through the similar electrophysical response to drugs like in vivo organs. The advancements made in using hydrogel scaffolds in combination with custom bioink has been used to fabricate a myocardium capable of full contraction [87]. Bioprinting has also been used to fabricate liver-on-a-chip devices. The use of bioprinting has assisted in extending the liver functions for weeks [88]. As a result, better models have been created to study drug hepatotoxicity and its clearance through the liver [89]. A fairly newer field of application for bioprinting is its use for developing placenta-on-a-chip. A recent study bioprinted a placental extracellular matrix “to model the fetal invasion of maternal vasculature” [90]. Bioprinting has advanced patient-specific OOCs as shown by the development of a bone-on-a-chip device derived from patients to assist in personalized therapy [91]. The potential of BOCs has also been shown using bioprinting with the fabrication of systems using heart-, liver-, and lung-on-a-chip for drug discovery and development [80,92]. Bioprinting has been combined with other existing fabrication techniques to achieve more accurate and complex models. For example, soft lithography and bioprinting were used together to produce a BOC consisting of a liver, heart, and lung [92]. However, bioprinting still faces many challenges when attempting to fabricate OOCs. Bioprinting struggles to display certain physiologic properties due to its inability to print small feature sizes with high enough resolution when using droplet-based methods (e.g., inkjet) [93]. As a result, certain OOCs like kidney-on-a-chip devices fail to mimic essential components of their in vivo counterpart [93]. In addition to this, bioprinting has struggled with fabricating super soft materials which are key in the development of brain- and lung-on-a-chip devices [94].

### 3.4. Injection Molding

**Figure 7:**
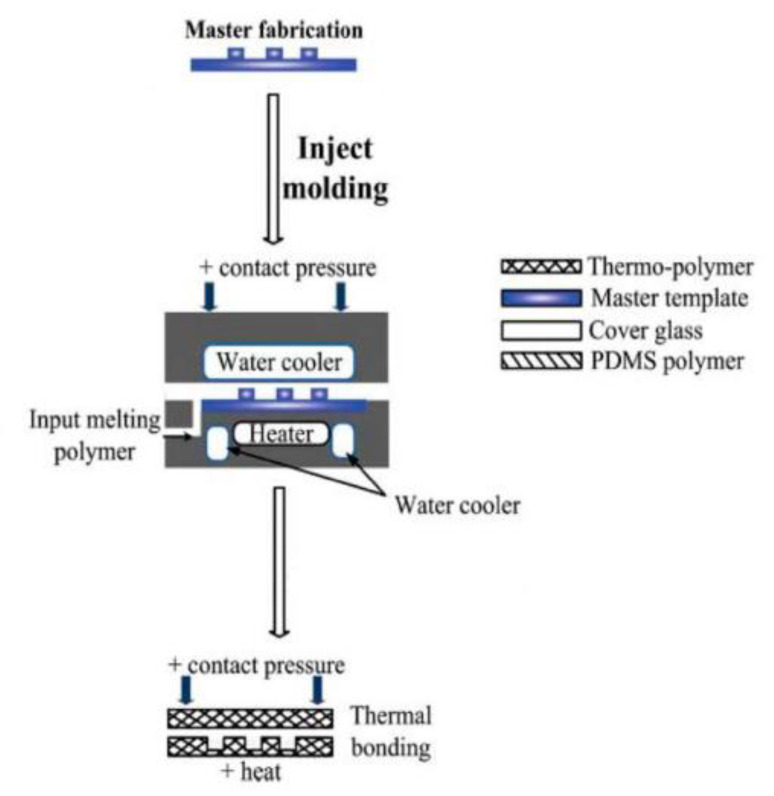
A typical injection molding process (reprinted with permission from Gale et al.; copyright (2018) [29]). A master is placed within a chamber and the injection material is melted. Upon the molds being compressed together, the material is injected into the mold cavity. After the mold cools off, it is removed from the mold cavity. [29].

Injection molding is a process that is broken down into the following four basic steps: (1) the material is melted, (2) the molds are compressed together, (3) the material is injected into the mold cavity, and (4) the mold is cooled and removed from the mold cavity (Figure 7) [29]. Injection molding is predominantly used during large-scale fabrication productions and focused on developing only the first element of OOCs (e.g., the microfluidic device) [58]. Injection molding was used to fabricate a liver-on-a-chip to study hypothermic storage [52]. While injection molding appears to be a simple and easy process to use, it requires familiarity and experience with it to be successfully performed at the micro level [58]. The primary disadvantages of injection molding have to do with the limited materials available to use and the mold features needing to be fairly simple (e.g., no undercuts) [95]. The main areas of innovation in injection molding are reducing the cost, reducing the time of the process, and improving the method to use other materials [29]. One method of innovation occurred by integrating complex fluid handling using interferometric sensors with the injection molding process [96]. Meanwhile in an attempt to reduce the cost and time associated with injection molding, 3D printing has been utilized to create quick and low-cost molds [97,98]. However, the high startup costs associated with injection molding has predominantly limited it to commercial applications requiring large-scale production as opposed to research development.

## 4. Future Challenges and Conclusion

To make further progress in the fabrication of OOCs, current hurdles need to be overcome. The utilization of PDMS or a newer material to achieve high bio-resistance while still functioning well with living cells and soft lithography is one step [100]. The automation of the various fabrication processes needs be achieved to create a standardized process across the field [59]. While CAD has been used in the field already, a broader application is required to assist in the optimization and standardization of the processes. This will assist in the modularization of the field allowing for quick and easy formation of BOCs by combining standardized pre-built OOCs to study all aspects of the human body [59]. In addition, the interconnections used in BOCs between the various organs need improvement to better mimic in vivo systems [59]. Another area of challenge is the fabrication and integration of the third and fourth elements (e.g., mechanisms for stimulus loading and sensors for monitoring) of OOCs mentioned earlier. Though these aspects are barely explored in this review, we note that it is an essential component to observing and studying OOCs and BOCs. The challenge of developing sensors at the microscale level with the necessary sensitivity and control has plagued the field of microfluidics for years. Another challenge of embedding sensors is to ensure that the sensors incorporated into or open to the culture channel are not toxic to the tissues and cells [19]. The techniques used in MEMS could prove helpful in the integration of sensors [19]. Ultimately, as these challenges are overcome, OOCs will become more complex and dynamic under control, allowing for the potential replacement of animal studies and helping advance drug discovery and development.

With the widely growing interest in the field of OOCs, it has experienced a mix of technological innovations and stagnations as techniques (e.g., bioprinting has allowed for more complex devices to be built but the minimum feature size and resolution attainable over the last decade has hardly changed). New methods have been established to allow more complex and in vitro models to be formed ranging from nanoimprint soft lithography to bioprinting. The combination of these methods has shown potential to further the fabrication process of OOCs. In addition, focus has been dedicated to lowering the cost of fabricating OOCs and using new materials like custom bioinks. However, the minimum feature size has changed little over this time requiring complex and expensive processes and machinery to accomplish sub micrometer structures. Laser-induced methods provide precise and high-resolution options to producing device, but their complexity and high costs due to the required machinery prevent its wide application. Meanwhile, soft lithography remains the foundation of fabrication techniques for OOCs and has been used for nearly every type of OOC. Still, the resolution and material limitations have slowed its innovation. The various methods of 3D printing in combination with bioprinting has become a nearly “skill-less” fabrication process with low cost, great automation, and fast production [58]. Yet, the high costs associated with printers capable of microscale fabrication and the challenges of developing custom bioink for each type of printer has stifled further innovation [58]. The use of injection molding has great promise when producing OOCs at the commercial level, but the existing methods used are too time consuming and costly to prove efficient at the research and development level. As the field of OOCs continue to grow further, the fabrication techniques will be innovated to create better models with faster development time, lower costs, and standardized modularized devices.

## Figures and Tables

**Table 1:** A brief overview of the fabrication methods used to create OOCs.

Fabrication Technique	Description	Pros and Cons	Materials	Application(s)
Photolithography	Patterning photoresist onto a substrate using light	Pros: -Well known technique -Sub-micrometer features possible Cons: -Limited by the materials available to use -Inability to allow gas to permeate -High costs -Low throughput	Silicon or silicon-based glasses with photoresist	Creating the master mold; Lung-on-a-chip [19]; Liver-on-a-chip [19];
Etching	Removing unprotected material away from the device using chemicals	Pros: -Well known technique -Sub-micrometer features possible -Various etching techniques that can be used for different applications Cons: -Etchant can damage favorable polymers -High costs -Low throughput	Silicon or silicon-based glasses with photoresist; Metals (e.g., Al, Cr, Au, Pt, Ti, etc.);	Creating the master mold; Porous membranes [30,31];
Laser-Induced Methods	Use of a laser to pattern a device (e.g., laser machining)	Pros: -Small feature sizes capable Cons: -Require high technical knowledge -High costs	Metals; Glass; Hydrogels;	Eye-on-a-chip [32]; Liver-on-a-chip [14];
Soft Lithography	Cure soft elastomers on a master mold	Pros: -Low cost -Easy to use -High compatibility with various materials Cons: -Low bio-resistance associated with the materials used -Requires masks and dedicated equipment -Requires familiarity with technique and manual operations	Soft elastomers (e.g., PDMS, PU, polyimide)	Lung-on-a-chip [4,33]; Liver-on-a-chip [14,34,35]; Gut-on-a-chip [35,36,37]; Retina-on-a-chip [38]; Most suited for large scale production; Porous membranes for OOCs [39];
Stereolithography	Using a UV laser to cure fluid resin layer-by-layer	Pros: -Most widely used 3D printing technique -Highly commercialized -High resolution for printers-Low cost Cons: -Low resolution compared to other non-3D printed techniques -Issues with printing microtissues	Photocurable resin/polymers; Bio-resins (e.g., PEGDA)	Lung-on-a-chip [40]; Vascular-on-a-chip [41]; General cell culture-on-a-chip [42];
Extrusion-Based	Using extrusion to place melted material layer-by-layer	Pros: -Able to fabricate the microfluidic chip and microtissues -Ability to print highly viscous bioinks -Continuous printing -Low costs -Easy to fabricate with -Faster than conventional methods (e.g., photolithography and etching) Cons: -Extrusion can place high stress on cells/tissues -High initial costs of the machines	Thermoplastics (e.g., ABS, PLA, polyamide, etc.); Bioinks;	Liver-on-a-chip [43]; Tumor-on-a-chip [44]; Kidney-on-a-chip [45]; Lung-on-a-chip [46]; Bone-on-a-chip [47,48];
Inkjet	Curing picolitre droplets layer-by-layer	Pros: -Able to fabricate the microfluidic chip and microtissues -Low costs -High quality and accuracy -Fast build time -Compatible with various materials Cons: -Has not been widely explored -Removal of support structures is challenging	Photocurable resin/polymers; Custom bioinks;	Liver-on-a-chip [49]; Co-cultured microfluidic environments [50];
Bioprinting	Printings cells and biomaterials into structures using existing 3D printing methods	Pros: -Versatility -Assembly-free process -Compatible with various materials -Incorporation of live cells in the ink Cons: -Low resolution -Not compatible with super soft materials -Slower than other methods	Hydrogels; Photocurable resin/polymers; Custom bioinks;	Vascular-networks-on-a-chip [51];
Injection Molding	Injecting a melted material into a mold cavity and cooling it	Pros: -Great for large scale production Cons: -Requires high technical knowledge -Limited materials available -Requires a fairly simple device -High startup costs limits it to commercial applications	Thermoplastics (e.g., ABS, PLA, polyamide, etc.);	Liver-on-a-chip [52];

**Table 2:** A list of potential organ models and their applications fabricated from various 3D printing techniques.

3D Printing Technique	Organ Model	Application
Stereolithography	Lung	Study inflammation-induced thrombosis on a lung-alveolus-on-a-chip [40]
Stereolithography	Vascular	Fabrication of 3D structures containing perfusion networks for a vascular system [41]
Stereolithography	General cell culture	Supporting multicellular spheroid culture via a single microfluidic device [42]
Extrusion-Based	Liver	One-step fabrication of liver-on-a-chip for metabolism and drug sensitivity studies [43]
Extrusion-Based	Tumor	Tumor model for in vitro pharmacokinetics studies [44]
Extrusion-Based	Bone, cartilage, & muscle	Produce human-scale tissue constructs with structural integrity [48]
Extrusion-Based	Kidney	Advanced human kidney tissues models for epithelial barrier disruption study [45]
Extrusion-Based	Lung	Asthmatic airway inflammation and allergen-induced asthma exacerbation model [46]
Extrusion-Based	Bone	Bone-on-a-chip for bone metastasis study of breast cancer cells [47]
Inkjet	Liver	Multiple cell patterning for drug metabolism and diffusion studies [49]
Inkjet	General cell culture	Simple to use method for long term culture of hydrogel encapsulated cell constructs [50]
Bioprinting	Vascular Networks	Fabrication of hydrogel microchannels to serve as vascular networks [51,58]

## References

[1] Wouters O.J., McKee M., Luyten J. (2020). Estimated Research and Development Investment Needed to Bring a New Medicine to Market, 2009–2018. JAMA.

[2] Morgan S., Grootendorst P., Lexchin J., Cunningham C., Greyson D. (2011). The cost of drug development: A systematic review. Health Policy.

[3] DiMasi J.A., Grabowski H.G., Hansen R.W. (2016). Innovation in the pharmaceutical industry: New estimates of R&D costs. J. Health Econ..

[4] Huh D., Matthews B.D., Mammoto A., Montoya-Zavala M., Hsin H.Y., Ingber D.E. (2010). Reconstituting Organ-Level Lung Functions on a Chip. Science.

[5] Astashkina A., Mann B., Grainger D.W. (2012). A critical evaluation of in vitro cell culture models for high-throughput drug screening and toxicity. Pharmacol. Ther..

[6] Wnorowski A., Yang H., Wu J.C. (2019). Progress, obstacles, and limitations in the use of stem cells in organ-on-a-chip models. Adv. Drug Deliv. Rev..

[7] Davila J.C., Rodriguez R.J., Melchert R.B., Acosta D. (1998). Predictive value of in vitro model systems in toxicology. Annu. Rev. Pharmacol. Toxicol..

[8] Huh D., Kim H.J., Fraser J.P., Shea D.E., Khan M., Bahinski A., Hamilton G.A., Ingber D.E. (2013). Microfabrication of human organs-on-chips. Nat. Protoc..

[9] Cook D., Brown D., Alexander R., March R., Morgan P., Satterthwaite G., Pangalos M.N. (2014). Lessons learned from the fate of AstraZeneca’s drug pipeline: A five-dimensional framework. Nat. Rev. Drug Discov..

[10] Miller P.G., Shuler M.L. (2016). Design and demonstration of a pumpless 14 compartment microphysiological system. Biotechnol. Bioeng..

[11] Hay M., Thomas D.W., Craighead J.L., Economides C., Rosenthal J. (2014). Clinical development success rates for investigational drugs. Nat. Biotechnol..

[12] Wong C.H., Siah K.W., Lo A.W. (2018). Estimation of clinical trial success rates and related parameters. Biostatistics.

[13] Takebe T., Zhang B., Radisic M. (2017). Synergistic Engineering: Organoids Meet Organs-on-a-Chip. Cell Stem Cell.

[14] Bavli D., Prill S., Ezra E., Levy G., Cohen M., Vinken M., Vanfleteren J., Jaeger M., Nahmias Y. (2016). Real-time monitoring of metabolic function in liver-on-chip microdevices tracks the dynamics of mitochondrial dysfunction. Proc. Natl. Acad. Sci. USA.

[15] Lee J.S., Romero R., Han Y.M., Kim H.C., Kim C.J., Hong J.-S., Huh D. (2016). Placenta-on-a-chip: A novel platform to study the biology of the human placenta. J. Matern. Fetal. Neonatal. Med..

[16] Ahn S.I., Sei Y.J., Park H.-J., Kim J., Ryu Y., Choi J.J., Sung H.-J., MacDonald T.J., Levey A.I., Kim Y. (2020). Microengineered human blood–brain barrier platform for understanding nanoparticle transport mechanisms. Nat. Commun..

[17] Esch M.B., King T.L., Shuler M.L. (2011). The Role of Body-on-a-Chip Devices in Drug and Toxicity Studies. Annu. Rev. Biomed. Eng..

[18] Sung J.H., Esch M.B., Prot J.-M., Long C.J., Smith A., Hickman J.J., Shuler M.L. (2013). Microfabricated mammalian organ systems and their integration into models of whole animals and humans. Lab Chip.

[19] Yang Q., Lian Q., Xu F. (2017). Perspective: Fabrication of integrated organ-on-a-chip via bioprinting. Biomicrofluidics.

[20] Vogelaar L., Barsema J.N., van Rijn C.J.M., Nijdam W., Wessling M. (2003). Phase Separation Micromolding—PSμM. Adv. Mater..

[21] Ren K., Chen Y., Wu H. (2014). New materials for microfluidics in biology. Curr. Opin. Biotechnol..

[22] Thorsen T., Maerkl S.J., Quake S.R. (2002). Microfluidic Large-Scale Integration. Science.

[23] Quake S.R., Scherer A. (2000). From Micro- to Nanofabrication with Soft Materials. Science.

[24] Bjorkholm J. (2001). EUV Lithography—The Successor to Optical Lithography?. Intel. Technol. J..

[25] Benschop J., Banine V., Lok S., Loopstra E. (2008). Extreme ultraviolet lithography: Status and prospects. J. Vac. Sci. Technol. BMicroelectron. Nanometer Struct. Process. Meas. Phenom..

[26] Xi Y., Zhang W., Fan Z., Ma Q., Wang S., Ma D., Jiang Z., Li H., Zhang Y. (2018). A facile synthesis of silicon nanowires/micropillars structure using lithography and metal-assisted chemical etching method. J. Solid State Chem..

[27] Harriott L.R. (2001). Limits of lithography. Proc. IEEE.

[28] Kolodziej C.M., Maynard H.D. (2012). Electron-Beam Lithography for Patterning Biomolecules at the Micron and Nanometer Scale. Chem. Mater..

[29] Gale B.K., Jafek A.R., Lambert C.J., Goenner B.L., Moghimifam H., Nze U.C., Kamarapu S.K. (2018). A Review of Current Methods in Microfluidic Device Fabrication and Future Commercialization Prospects. Inventions.

[30] Abhyankar V.V., Wu M., Koh C.-Y., Hatch A.V. (2016). A Reversibly Sealed, Easy Access, Modular (SEAM) Microfluidic Architecture to Establish In Vitro Tissue Interfaces. PLoS ONE.

[31] Pasman T., Grijpma D., Stamatialis D., Poot A. (2018). Flat and microstructured polymeric membranes in organs-on-chips. J. R. Soc. Interface.

[32] Chan Y.K., Sy K.H.S., Wong C.Y., Man P.K., Wong D., Shum H.C. (2015). In Vitro Modeling of Emulsification of Silicone Oil as Intraocular Tamponade Using Microengineered Eye-on-a-Chip. Investig. Ophthalmol. Vis. Sci..

[33] Yang X., Li K., Zhang X., Liu C., Guo B., Wen W., Gao X. (2018). Nanofiber membrane supported lung-on-a-chip microdevice for anti-cancer drug testing. Lab Chip.

[34] Banaeiyan A.A., Theobald J., Paukštyte J., Wölfl S., Adiels C.B., Goksör M. (2017). Design and fabrication of a scalable liver-lobule-on-a-chip microphysiological platform. Biofabrication.

[35] Choe A., Ha S.K., Choi I., Choi N., Sung J.H. (2017). Microfluidic Gut-liver chip for reproducing the first pass metabolism. Biomed. Microdevices.

[36] Jalili-Firoozinezhad S., Prantil-Baun R., Jiang A., Potla R., Mammoto T., Weaver J.C., Ferrante T.C., Kim H.J., Cabral J.M.S., Levy O. (2018). Modeling radiation injury-induced cell death and countermeasure drug responses in a human Gut-on-a-Chip. Cell Death Dis..

[37] Villenave R., Wales S.Q., Hamkins-Indik T., Papafragkou E., Weaver J.C., Ferrante T.C., Bahinski A., Elkins C.A., Kulka M., Ingber D.E. (2017). Human Gut-On-A-Chip Supports Polarized Infection of Coxsackie B1 Virus In Vitro. PLoS ONE.

[38] Dodson K.H., Echevarria F.D., Li D., Sappington R.M., Edd J.F. (2015). Retina-on-a-chip: A microfluidic platform for point access signaling studies. Biomed. Microdevices.

[39] Kim Y.T., Castro K., Bhattacharjee N., Folch A. (2018). Digital Manufacturing of Selective Porous Barriers in Microchannels Using Multi-Material Stereolithography. Micromachines.

[40] Jain A., Barrile R., Van der Meer A., Mammoto A., Mammoto T., De Ceunynck K., Aisiku O., Otieno M., Louden C., Hamilton G. (2018). Primary Human Lung Alveolus-on-a-chip Model of Intravascular Thrombosis for Assessment of Therapeutics. Clin. Pharmacol. Ther..

[41] Zhang R., Larsen N.B. (2017). Stereolithographic hydrogel printing of 3D culture chips with biofunctionalized complex 3D perfusion networks. Lab Chip.

[42] Ong L.J.Y., Islam A., DasGupta R., Iyer N.G., Leo H.L., Toh Y.-C. (2017). A 3D printed microfluidic perfusion device for multicellular spheroid cultures. Biofabrication.

[43] Lee H., Cho D.-W. (2016). One-step fabrication of an organ-on-a-chip with spatial heterogeneity using a 3D bioprinting technology. Lab Chip.

[44] Chang R., Nam J., Sun W. (2008). Direct Cell Writing of 3D Microorgan for In Vitro Pharmacokinetic Model. Tissue Eng. Part C Methods.

[45] Homan K.A., Kolesky D.B., Skylar-Scott M.A., Herrmann J., Obuobi H., Moisan A., Lewis J.A. (2016). Bioprinting of 3D Convoluted Renal Proximal Tubules on Perfusable Chips. Sci. Rep..

[46] Horváth L., Umehara Y., Jud C., Blank F., Petri-Fink A., Rothen-Rutishauser B. (2015). Engineering an in vitro air-blood barrier by 3D bioprinting. Sci. Rep..

[47] Hao S., Ha L., Cheng G., Wan Y., Xia Y., Sosnoski D.M., Mastro A.M., Zheng S.-Y. (2018). A Spontaneous 3D Bone-On-a-Chip for Bone Metastasis Study of Breast Cancer Cells. Small.

[48] Kang H.-W., Lee S.J., Ko I.K., Kengla C., Yoo J.J., Atala A. (2016). A 3D bioprinting system to produce human-scale tissue constructs with structural integrity. Nat. Biotechnol..

[49] Zhang J., Chen F., He Z., Ma Y., Uchiyama K., Lin J.-M. (2016). A novel approach for precisely controlled multiple cell patterning in microfluidic chips by inkjet printing and the detection of drug metabolism and diffusion. Analyst.

[50] Hamid Q., Wang C., Snyder J., Williams S., Liu Y., Sun W. (2015). Maskless fabrication of cell-laden microfluidic chips with localized surface functionalization for the co-culture of cancer cells. Biofabrication.

[51] Bertassoni L.E., Cecconi M., Manoharan V., Nikkhah M., Hjortnaes J., Cristino A.L., Barabaschi G., Demarchi D., Dokmeci M.R., Yang Y. (2014). Hydrogel bioprinted microchannel networks for vascularization of tissue engineering constructs. Lab Chip.

[52] Gröger M., Dinger J., Kiehntopf M., Peters F.T., Rauen U., Mosig A.S. (2018). Preservation of Cell Structure, Metabolism, and Biotransformation Activity of Liver-On-Chip Organ Models by Hypothermic Storage. Adv. Healthc. Mater..

[53] Nawroth J.C., Scudder L.L., Halvorson R.T., Tresback J., Ferrier J.P., Sheehy S.P., Cho A., Kannan S., Sunyovszki I., Goss J.A. (2018). Automated fabrication of photopatterned gelatin hydrogels for organ-on-chips applications. Biofabrication.

[54] Lin Z.J., Xu J., Song Y.P., Li X.L., Wang P., Chu W., Wang Z.H., Cheng Y. (2020). Freeform Microfluidic Networks Encapsulated in Laser-Printed 3D Macroscale Glass Objects. Adv. Mater. Technol..

[55] Cooksey G.A., Atencia J. (2014). Pneumatic valves in folded 2D and 3D fluidic devices made from plastic films and tapes. Lab Chip.

[56] Rajan S.A.P., Aleman J., Wan M., Pourhabibi Zarandi N., Nzou G., Murphy S., Bishop C.E., Sadri-Ardekani H., Shupe T., Atala A. (2020). Probing prodrug metabolism and reciprocal toxicity with an integrated and humanized multi-tissue organ-on-a-chip platform. Acta Biomater..

[57] Rajan S.A.P., Skardal A., Hall A.R. (2020). Multi-Domain Photopatterned 3D Tumor Constructs in a Micro-Physiological System for Analysis, Quantification, and Isolation of Infiltrating Cells. Adv. Biosyst..

[58] Ho C.M., Ng S.H., Li K.H., Yoon Y.J. (2015). 3D printed microfluidics for biological applications. Lab Chip.

[59] Wang Z., Samanipour R., Kim K., Jo H., Jun H.-W., Shin J., Lee S. (2016). Organ-on-a-Chip Platforms for Drug Screening and Tissue Engineering. Biomedical Engineering: Frontier Research and Converging Technologies.

[60] Duffy D.C., McDonald J.C., Schueller O.J.A., Whitesides G.M. (1998). Rapid Prototyping of Microfluidic Systems in Poly(dimethylsiloxane). Anal. Chem..

[61] Weibel D.B., Diluzio W.R., Whitesides G.M. (2007). Microfabrication meets microbiology. Nat. Rev. Microbiol..

[62] Whitesides G.M., Ostuni E., Takayama S., Jiang X., Ingber D.E. (2001). Soft lithography in biology and biochemistry. Annu. Rev. Biomed. Eng..

[63] Lee J.N., Jiang X., Ryan D., Whitesides G.M. (2004). Compatibility of mammalian cells on surfaces of poly(dimethylsiloxane). Langmuir.

[64] Gates B.D., Xu Q., Stewart M., Ryan D., Willson C.G., Whitesides G.M. (2005). New Approaches to Nanofabrication:  Molding, Printing, and Other Techniques. Chem. Rev..

[65] Rolland J.P., Hagberg E.C., Denison G.M., Carter K.R., De Simone J.M. (2004). High-Resolution Soft Lithography: Enabling Materials for Nanotechnologies. Angew. Chem. Int. Ed..

[66] Rolland J.P., Van Dam R.M., Schorzman D.A., Quake S.R., DeSimone J.M. (2004). Solvent-Resistant Photocurable “Liquid Teflon” for Microfluidic Device Fabrication. J. Am. Chem. Soc..

[67] Blundell C., Yi Y.-S., Ma L., Tess E.R., Farrell M.J., Georgescu A., Aleksunes L.M., Huh D. (2018). Placental Drug Transport-on-a-Chip: A Microengineered In Vitro Model of Transporter-Mediated Drug Efflux in the Human Placental Barrier. Adv. Healthc. Mater..

[68] Wang Y., Wang L., Zhu Y., Qin J. (2018). Human brain organoid-on-a-chip to model prenatal nicotine exposure. Lab Chip.

[69] Hassell B.A., Goyal G., Lee E., Sontheimer-Phelps A., Levy O., Chen C.S., Ingber D.E. (2017). Human Organ Chip Models Recapitulate Orthotopic Lung Cancer Growth, Therapeutic Responses, and Tumor Dormancy In Vitro. Cell Rep..

[70] Sticker D., Rothbauer M., Lechner S., Hehenberger M.T., Ertl P. (2015). Multi-layered, membrane-integrated microfluidics based on replica molding of a thiol-ene epoxy thermoset for organ-on-a-chip applications. Lab Chip.

[71] Ugolini G.S., Visone R., Redaelli A., Moretti M., Rasponi M. (2017). Generating Multicompartmental 3D Biological Constructs Interfaced through Sequential Injections in Microfluidic Devices. Adv. Healthc. Mater..

[72] Cross G.L.W. (2006). The production of nanostructures by mechanical forming. J. Phys. D Appl. Phys..

[73] https://global.canon/en/news/2015/feb23e3.html.

[74] Xia Q., Pease R.F. (2015). Nanoimprint lithography 20 years on. Nanotechnology.

[75] Hull C.W. (1984). Apparatus for Production of Three-Dimensional Objects by Stereolithography. U.S. Patent.

[76] Avci H., Doğan Güzel F., Erol S., Akpek A. (2017). Recent Advances in Organ-on-a-chip Technologies and Future Challenges: A Review. Turk. J. Chem..

[77] Mandrycky C., Wang Z., Kim K., Kim D.H. (2016). 3D bioprinting for engineering complex tissues. Biotechnol. Adv..

[78] Tumbleston J.R., Shirvanyants D., Ermoshkin N., Janusziewicz R., Johnson A.R., Kelly D., Chen K., Pinschmidt R., Rolland J.P., Ermoshkin A. (2015). Continuous liquid interface production of 3D objects. Science.

[79] Gong H., Bickham B.P., Woolley A.T., Nordin G.P. (2017). Custom 3D printer and resin for 18 μm × 20 μm microfluidic flow channels. Lab Chip.

[80] Lee S.H., Sung J.H. (2018). Organ-on-a-Chip Technology for Reproducing Multiorgan Physiology. Adv. Healthc. Mater..

[81] Lee M.P., Cooper G.J.T., Hinkley T., Gibson G.M., Padgett M.J., Cronin L. (2015). Development of a 3D printer using scanning projection stereolithography. Sci. Rep..

[82] Zein I., Hutmacher D.W., Tan K.C., Teoh S.H. (2002). Fused deposition modeling of novel scaffold architectures for tissue engineering applications. Biomaterials.

[83] Au A.K., Huynh W., Horowitz L.F., Folch A. (2016). 3D-Printed Microfluidics. Angew. Chem. Int. Ed..

[84] Lind J.U., Busbee T.A., Valentine A.D., Pasqualini F.S., Yuan H., Yadid M., Park S.J., Kotikian A., Nesmith A.P., Campbell P.H. (2017). Instrumented cardiac microphysiological devices via multimaterial three-dimensional printing. Nat. Mater..

[85] Boland T.W., William C., Xu T. (2003). Ink-Jet Printing of Viable Cells. U.S. Patent.

[86] Malda J., Visser J., Melchels F.P., Jüngst T., Hennink W.E., Dhert W.J.A., Groll J., Hutmacher D.W. (2013). 25th Anniversary Article: Engineering Hydrogels for Biofabrication. Adv. Mater..

[87] Zhang Y.S., Arneri A., Bersini S., Shin S.-R., Zhu K., Goli-Malekabadi Z., Aleman J., Colosi C., Busignani F., Dell’Erba V. (2016). Bioprinting 3D microfibrous scaffolds for engineering endothelialized myocardium and heart-on-a-chip. Biomaterials.

[88] Kizawa H., Nagao E., Shimamura M., Zhang G., Torii H. (2017). Scaffold-free 3D bio-printed human liver tissue stably maintains metabolic functions useful for drug discovery. Biochem. Biophys. Rep..

[89] Mittal R., Woo F.W., Castro C.S., Cohen M.A., Karanxha J., Mittal J., Chhibber T., Jhaveri V.M. (2019). Organ-on-chip models: Implications in drug discovery and clinical applications. J. Cell. Physiol..

[90] Kuo C.-Y., Guo T., Cabrera-Luque J., Arumugasaamy N., Bracaglia L., Garcia-Vivas A., Santoro M., Baker H., Fisher J., Kim P. (2018). Placental basement membrane proteins are required for effective cytotrophoblast invasion in a three-dimensional bioprinted placenta model. J. Biomed. Mater. Res. Part A.

[91] Arrigoni C., Gilardi M., Bersini S., Candrian C., Moretti M. (2017). Bioprinting and Organ-on-Chip Applications Towards Personalized Medicine for Bone Diseases. Stem Cell Rev. Rep..

[92] Skardal A., Murphy S.V., Devarasetty M., Mead I., Kang H.-W., Seol Y.-J., Shrike Zhang Y., Shin S.-R., Zhao L., Aleman J. (2017). Multi-tissue interactions in an integrated three-tissue organ-on-a-chip platform. Sci. Rep..

[93] Murphy S.V., Atala A. (2014). 3D bioprinting of tissues and organs. Nat. Biotechnol..

[94] Tan Z., Parisi C., Di Silvio L., Dini D., Forte A.E. (2017). Cryogenic 3D Printing of Super Soft Hydrogels. Sci. Rep..

[95] Wu J., Gu M. (2011). Microfluidic sensing: State of the art fabrication and detection techniques. J. Biomed. Opt..

[96] Szydzik C., Gavela A.F., Herranz S., Roccisano J., Knoerzer M., Thurgood P., Khoshmanesh K., Mitchell A., Lechuga L.M. (2017). An automated optofluidic biosensor platform combining interferometric sensors and injection moulded microfluidics. Lab Chip.

[97] Lin T.-Y., Do T., Kwon P., Lillehoj P.B. (2017). 3D printed metal molds for hot embossing plastic microfluidic devices. Lab Chip.

[98] Vereshchagina E., Andreassen E., Gaarder R., Mielnik M. Synergy of 3D printing and injection molding: A new prototyping method for rapid design optimization and manufacturing of microfluidic devices. Proceedings of the 21st International Conference on Miniaturized Systems and Life Sciences.

[99] Liu W., Zhang Y.S., Heinrich M.A., De Ferrari F., Jang H.L., Bakht S.M., Alvarez M.M., Yang J., Li Y.-C., Trujillo-de Santiago G. (2017). Rapid Continuous Multimaterial Extrusion Bioprinting. Adv. Mater..

[100] Berthier E., Young E.W.K., Beebe D. (2012). Engineers are from PDMS-land, Biologists are from Polystyrenia. Lab Chip.

